# On the Diagnostic Value of the Symptoms Commonly Regarded as Indicative of Pulmonary Cavities

**Published:** 1857-06

**Authors:** 


*On the Diagnostic Value of the Symptoms commonly regarded as Indicative of Pulmonary Cavities.* By Dr. N. Friedreich. (Verhoddlungen der Physikalisch. Medicinischer Gesellsschaft in Wurzburg. Siebenter Band, Heft 1.)

The cracked-pot sound, the tympanitic percussion-sound, the amphoric and metallic respiratory sounds, are in this paper examined in relation to the diagnosis of pulmonary cavities. We recently drew attention to Professor Bennett's observations on the occurrence of the cracked-pot sound in various conditions unconnected with cavities. Dr. Cockle has also * shown that it may occur in cases of simple bronchitis. Dr. Feiedreich gives three cases of pleurisy in which this sound was met with. In the first (a man, aged twenty-two) it occurred in the left infra-clavicular region, at the time when the effusion on the same side was receding, and it lasted until its complete absorption. In the second (a man, aged twenty-two), the sound occurred from the commencement of the affection, and whether the nose and mouth were open or closed, in the left infra-clavicular space, as far as the third rib, to which the pleuritic effusion reached. It disappeared before any change in the exudation was perceived. In the third case (a man, aged twenty-twenty-three), the *bruit de pot fêlé* was produced, the mouth and nose being open, at the upper left side, down to the third rib, at which point the effusion commenced. The patient was still under observation when the paper was written. With regard to the occurrence of the sound in healthy subjects, Dr. Friedreich has failed to discover it in the adult, but on examining forty-six children under fourteen years of age, he met with it twenty-six times—fourteen times audible on both sides anteriorly, but only in five equally loud—in the other cases, generally louder on the left than the right side, and only twice louder on the right than the left. In explaining the production of the cracked-pot sound, Dr. Friedreich opposes the theory that it is due to air being forcibly expelled through the glottis, because on applying the stethoscope to the larynx, while another person produces the sound, no indication of its formation at the glottis is obtained. In bronchitis and

* Asssociation Medical Journal, July, 1856.

early infancy he believes the production of the sound to be due to the compression of the smaller bronchi during the act of percussion. He adopts Skoda's theory of its production in phthisis, while in pleurisy he attributes it to compression of the pulmonary tissue by the exudation, and the forcible expulsion through the smaller bronchi of the air contained in them, when percussion is employed.

